# Transcriptome Differences in Porcine Alveolar Macrophages from Tongcheng and Large White Pigs in Response to Highly Pathogenic Porcine Reproductive and Respiratory Syndrome Virus (PRRSV) Infection

**DOI:** 10.3390/ijms18071475

**Published:** 2017-07-12

**Authors:** Wan Liang, Likai Ji, Yu Zhang, Yueran Zhen, Qingde Zhang, Xuewen Xu, Bang Liu

**Affiliations:** 1 Key Laboratory of Agricultural Animal Genetics, Breeding and Reproduction of Ministry of Education, Key Laboratory of Pig Genetics and Breeding of Ministry of Agriculture & College of Animal Science and Technology, Huazhong Agricultural University, Wuhan 430070, China; liangwan521521@163.com (W.L.); jilikai@webmail.hzau.edu.cn (L.J.); arthas_54@126.com (Y.Z.) zhenyueran@webmail.hzau.edu.cn (Y.Z.); 2 The Cooperative Innovation Center for Sustainable Pig Production, Wuhan 430070, China; 3 Laboratory Animal Center, College of Animal Science and Technology & Veterinary Medicine, Huazhong Agricultural University, Wuhan 430070, China; qdzhang@mail.hzau.edu.cn

**Keywords:** porcine reproductive and respiratory syndrome virus (PRRSV), challenge, Tongcheng pigs, large white pigs, porcine alveolar macrophages, transcriptome

## Abstract

Porcine reproductive and respiratory syndrome virus (PRRSV) is a single-stranded positive-sense RNA virus that can cause devastating reproductive failure and respiratory tract lesions, which has led to serious damage to the swine industry worldwide. Our previous studies have indicated that Tongcheng (TC) pigs, a Chinese local breed, have stronger resistance or tolerance to PRRSV infection than Large White (LW) pigs. This study aims to investigate their host transcriptome differences in porcine alveolar macrophages (PAMs) at 7 days post challenge. Transcriptome profiling of PAMs from PRRSV infected and control pigs of these two breeds were performed using RNA-sequencing. For both breeds, there were 1257 common differentially expressed genes (DEGs) in response to PRRSV infection, involving hepatic fibrosis/hepatic stellate cell activation, phospholipase C, and granulocyte adhesion and diapedesis pathways. For TC pig, 549 specific DEGs were identified, including VAV2, BCL2 and BAX, which were enriched in activation of leukocyte extravasation and suppression of apoptosis. While, 898 specific DEGs were identified in LW pigs, including GNAQ, GNB5, GNG2, CALM4 and RHOQ, which were involved in suppression of Gαq and PI3K-AKT signaling. This study provides an insight into the transcriptomic comparison of resistant and susceptible pigs to PRRSV infection. TC pigs may promote the extravasation and migration of leukocytes to defend against PRRSV infections and suppress apoptosis of the infected macrophages to increase antigen presentation, thereby reducing the lung lesions.

## 1. Introduction

As one of the most devastating diseases in the swine industry, porcine reproductive and respiratory syndrome (PRRS) has aroused much concern worldwide since its emergence. The disease is characterized by reproductive failure and severe respiratory symptoms and the causative agent is the porcine reproductive and respiratory syndrome virus (PRRSV), an enveloped, single-stranded positive-sense RNA virus, which belongs to the *Arteriviridae* family [1]. The primary target of PRRSV is porcine alveolar macrophages (PAMs) [2]. Once the host is infected by PRRSV, they will enter an acute viraemic stage, which lasts from one to four weeks, followed by a virus clearance stage with variable lengths of time, ranging from several weeks to 200 days [1,3,4]. As a positive-sense RNA virus, PRRSV evolves extremely rapidly and is divided into two main serotypes, North American and European, which display diverse pathogenic characteristics and host immune responses [5,6]. Due to the immune evasion and highly variable character of PRRSV, the development of commercial vaccines has so far been ineffective. In addition, other alternative strategies, such as breeding with highly resistant pigs, have been explored as a potential way to combat PRRS outbreak [7,8].

Previous studies of PRRS outbreak in pig populations, as well as numerous artificial infection experiments, have revealed that pig breed displayed variable clinical syndromes and virus clearance ability, indicating that host genetics contributed to tolerance variation [9]. To fully decipher the difference of immune response caused by different host genetics, Petry et al. conducted PRRSV artificial infection experiments in the Nebraska Index line and a commercial Hampshire × Duroc cross line, and classified the pigs into high response (HR) and low response (LR) groups according to the viremia in the early stages of infection [10]. Using an oligonucleotide microarray, Bates et al. compared the gene expression in both lung and bronchial lymph nodes of pigs from HR and LR groups, and identified four differentially expressed genes (DEGs), DEAD box RNA helicase 3 (DDX3), thymosin β-4 (Tβ4), acetylcholinesterase (ACHE) and X (inactive)-specific transcript (XIST) [11]. With the same panels of samples, Wysocki et al. further analyzed the involved pathways when there was tolerance to PRRSV by the long Pigoligoarray, and inferred that PRRSV infection prevented protective immune responses in HR pigs [12]. In 2010, another PRRSV artificial infection experiment, organized by the PRRS Host Genetics Consortium (PHGC), was conducted in a population of approximately 200 commercial pigs, which were classified into four phenotypic groups according to the viral levels in serum and levels of weight gain during acute infection stages [9]. With phenotypic divergent samples from the PHGC population, Arceo et al. identified transcripts differentially expressed in blood, which included the genes interferon-α 1 (IFNA1), major histocompatibility complex (MHC) class II, DQ α 1 (SLA-DQA1) and DR α (SLA-DRA) [13]. Jinyi et al. used a Chinese indigenous breed Dapulian (DPL) and Duroc × Landrace × Yorkshire (DLY) pigs to compare the breeds’ difference to highly pathogenic PRRSV (HP-PRRSV) infection [14]. They identified that the DPL exhibited mild clinical signs, and had a higher level of IFN-ω and lower level of IL-10 and TNF-α than DLY pigs [14]. All the studies were conducted with type 2 PRRSV and revealed that type 2 PRRSV infections could modulate the host’s innate and adaptive responses, but the responses varied among different hosts, indicating the complexity of the resistance genetic background.

In 2006, there was an epidemic of highly pathogenic PRRSV (HP-PRRSV) in China resulting in high morbidity and mortality [15]; however, some Chinese indigenous pig breeds displayed resistance to PRRSV infection, such as Tongcheng pigs [16] and DPL pigs [14]. We compared the artificial infection responses of Tongcheng (TC) and Large White (LW) pigs to HP-PRRSV and found that TC pigs had less severe symptoms and lower levels of viral load, indicating that TC pigs were more resistant to early HP-PRRSV infection [17]. We hypothesized that if the transcriptome differences could be compared, they might offer vital information for PRRSV immune regulatory mechanism. In this study, we employed the transcriptomic sequencing procedure in PAMs of TC and LW pigs infected with HP-PRRSV at 7 days post challenge (dpc) to assess the global gene expression at the initial stage of HP-PRRSV infection, which would give valuable insights into the mechanism that allow TC pigs to resist PRRSV infection.

## 2. Results

### 2.1. RNA Sequencing Data Mapping and Annotation

In total, 12 cDNA libraries from four groups (TC_Infection, TC_Control, LW_Infection, LW_Control, each group with three replications) were sequenced, which yielded in total 1771 million 100 bp paired-end clean reads, varying from 110 to 161 million for each sample (Table 1). Among the clean reads, more than 95.18% had quality scores at the Q20 level, and on average, approximately 74.82% clean reads were mapped to the reference genome (*Sus scrofa* 10.2.72) (Table 1).

**Figure 1:**
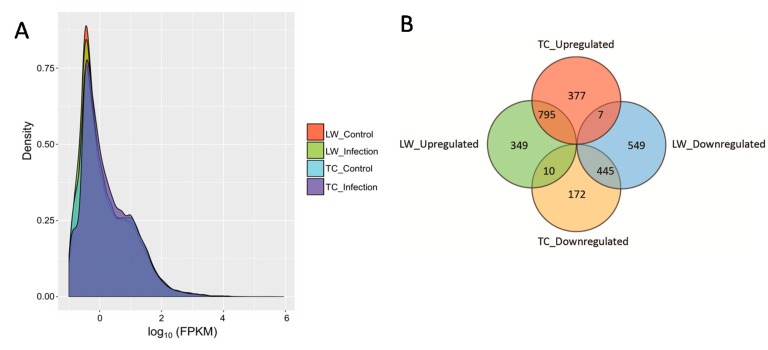
The density plot of genes’ log_10_ (fragments per kilobase of transcript per million mapped reads) (log_10_ (FPKM)) distribution visualized by CummeRbund and the Venn Diagram of differentially expressed genes (DEGs) distribution in each group. (**A**) The density plot of genes’ log_10_ (FPKM) distribution visualized by CummeRbund. The X-axis represents the log_10_ (FPKM) of all the genes. The Y-axis represents the genes’ distribution density. The four groups were shown by different colors; (**B**) the Venn Diagram of differentially expressed genes (DEGs) distribution in each group. The number of DEGs in Tongcheng (TC) and Large White (LW) pigs compared to their control groups (false discovery rates ≤ 0.05 and |log_2_ (Fold Change) ≥ 1|) were shown, and up and down regulated genes were separated. The numbers in overlapping areas represent DEGs shared between the two groups.

After assembling for each sample, Cuffdiff package in Cufflinks (available online: http://cole-trapnell-lab.github.io/cufflinks/), a program used to find significant changes in expression levels of genes and transcripts in RNA-Seq experiments, was used to calculate the fragments per kilobase of transcript per million mapped reads (FPKM) value of the four groups for each gene according to the pig 10.2 reference genome annotation (Table 1). With the FPKM threshold of 0.1, an aggregate of 15,364 expressed genes in TC, 15,241 in TC Control, 16,127 in LW and 15,621 in LW Control groups were detected, respectively. As shown in Figure 1A, the FPKM density in four groups displayed similar skewed distribution, approximately 53.61–55.53% genes were found to be lowly expressed (0.1 ≤ FPKM < 1) (Figure 1A).

### 2.2. Differentially Expressed Genes Calling and Validation by RT-qPCR and Western-Blotting

To quantify the basic genetic difference between TC and LW pigs, we analyzed the transcriptome difference between the two control groups. Compared with LW pigs, 333 DEGs were more expressed in TC pigs and 353 DEGs were less expressed in TC pigs (*p* value ≤ 0.05 and |log_2_ FC ≥ 1|) (Totally 686 DEGs). To analyze the transcriptome difference between healthy and infectious conditions, the infected groups were compared with their control groups of the same breed, respectively. Compared with the control group, 1179 genes were upregulated and 627 genes (in total, 1806 DEGs) were downregulated in TC pigs with PRRSV infection, and 1154 genes were upregulated and 1001 genes (in total, 2155 DEGs) were downregulated in LW pigs (*p* value ≤ 0.05 and |log_2_ FC ≥ 1|). TC and LW pigs shared 795 up-regulated genes and 445 downregulated genes response to PRRSV infection (Figure 1B). Meanwhile, both breeds had many unique DEGs in response to PRRSV infection. Particularly, 549 genes are uniquely downregulated in LW pigs with PRRSV infection, which is two times higher than the number of downregulated in TC pigs (172 genes) (Figure 1B). In addition, seven upregulated genes (*CDH1*, *IGKV-3*, *CCL17*, *SORCS1*, ENSSSCG00000024116, ENSSSCG00000011609, and ENSSSCG00000020872) in TC pigs were downregulated in LW pigs, and ten downregulated genes (*SCGB1A1*, *HIST1H2AK*, *CXCL13*, *PLBD1*, *CRISP3*, *SDC3*, *SLA-5*, *SLA-DRB4*, ENSSSCG00000001397, ENSSSCG00000017235) in TC pigs were upregulated in LW pigs.

**Figure 2:**
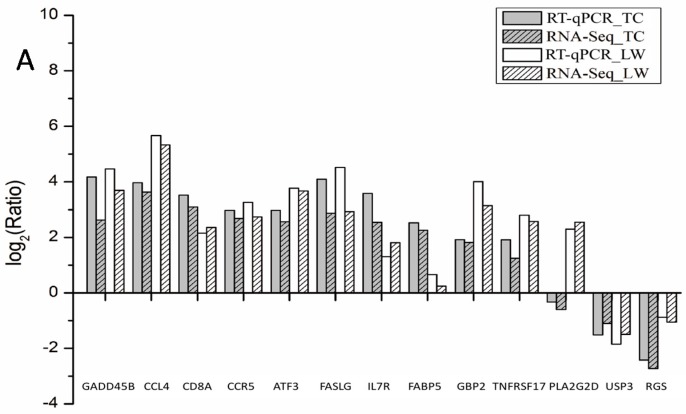

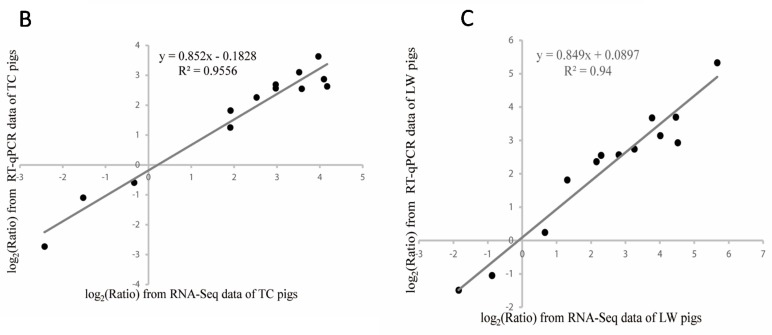
The RT-qPCR identification of randomly selected DEGs and correlation with RNA-seq data. (**A**) The RT-qPCR identification result of randomly selected DEGs. The X-axis is the name of genes and the Y-axis is the log_2_(Ratio) relative expression value; (**B**) the correlation of RT-qPCR and RNA-Seq of TC pigs; (**C**) the correlation of RT-qPCR and RNA-Seq of LW pigs.

To validate the expression pattern of DEGs identified from RNA-Seq, thirteen genes were selected to perform the real-time quantitative reverse transcription PCR (RT-qPCR) assays with the *SDHA* gene as internal control (Figure 2A). Among the selected DEGs, ten genes including *GADD45B*, *CCL4*, *CD8A*, *CCR5*, *ATF3*, *FASLG*, *IL7R*, *FABP5*, *GBP2*, *TNFRSF17*, displayed a consistent upregulation trend in response to PRRSV infection in both pig breeds, and USP33 and RGS4 were downregulated in PRRSV infection. Both RT-qPCR assay and RNA-seq revealed that PLA2G2D is slightly downregulated in TC pigs but significantly upregulated in LW pigs (Figure 2A). Although the extent of fold-change varied between RT-qPCR and RNA-Seq, the RT-qPCR assay confirmed the result of RNA-Seq, and both methods displayed strong correlation in both breeds (*R*^2^ = 0.9556 in TC pigs and 0.94 in LW pigs) (Figure 2B,C), indicating the high reliability of RNA-seq data.

**Figure 3:**
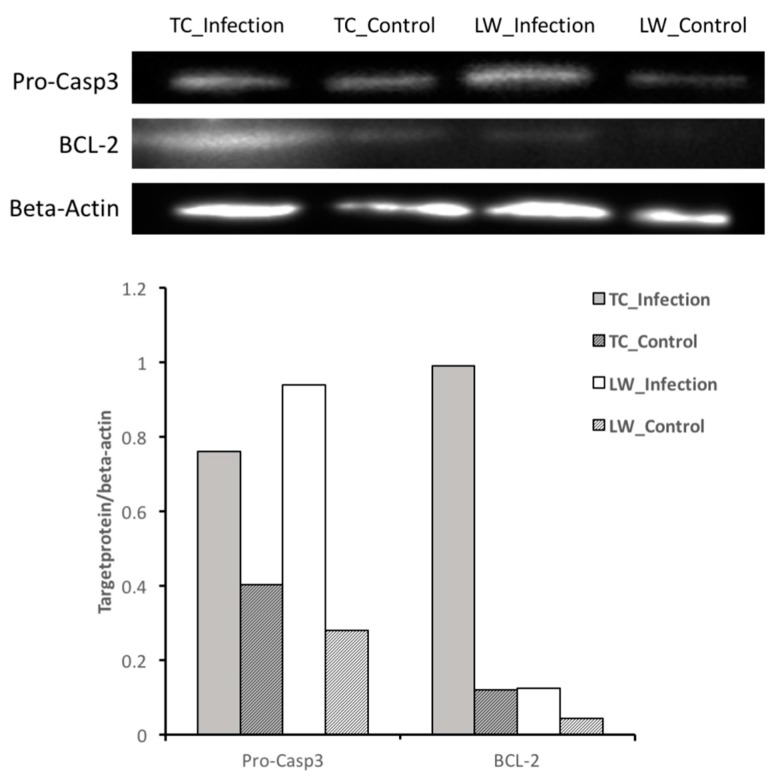
Detection of *pro-Casp3* and *BCL-2* protein expression by Western-blot assay. In the column, the X-axis is the protein’s name and the Y-axis is the ratio of the grey value of *pro-Casp3/β-Actin* or *BCL-2/Β-Actin*.

As described in the discussion below, there was a difference in apoptosis pathway between TC and LW pigs. To verify the genes in the pathway, the anti-apoptosis gene *BCL-2* and apoptotic protein precursor caspase 3 (*pro-Casp3*) were identified by Western-blot assay with β-actin as the housekeeping protein. TC and LW pigs had a low level of *BCL-2* and *pro-Casp3* in control groups (Figure 3). After infection, both *BCL-2* and *pro-Casp3* were up-regulated, while TC had a higher expression of *BCL-2* and lower expression of *pro-Casp3* than LW pigs.

### 2.3. The Basic Genetic Comparison of TC and LW Pigs Reveals Differences Among MHC Genes

To reveal the basic genetic differences of TC and LW pigs, the 686 DEGs between the two control groups were submitted to the Database for Annotation, Visualization, and Integrated Discovery (DAVID) [18] for gene ontology (GO) analysis, which identified 21 enriched biological process terms, including wound healing, immune response, inflammatory response, and cell migration. The Kyoto Encyclopedia of Genes and Genomes (KEGG) pathway analysis identified sixteen significant signaling pathways including cell adhesion molecules (CAMs) (including several *MHC* antigens, such as *SLA*, *SLA-6*, *SLA-DRB1*, *SLA-DRB4* higher in TC pigs, *SLA-DRB5* higher in LW pigs) and antigen processing and presentation (including *HSPA2* higher in TC pigs, *HSPA12A* higher in LW pigs). Among the 686 DEGs between two control groups, 269 DEGs of them remained differentially expressed after infection. The 269 DEGs were submitted to DAVID for GO and KEGG analysis, which identified 11 enriched biological process terms, including nucleosome assembly, *IL-2* secretion and negative regulation of protein phosphorylation. The KEGG pathway analysis identified 10 pathways including CAMs (including *SLA-DRB1*, *CLDN4*, *CLDN18*, *CD40LG*), further supporting the basic genetic difference of *MHC* antigens between breeds.

### 2.4. Host’s Response to PRRSV Infection Reveals the Activation of the Host Pathogen Recognition Receptors and the Production of Inflammatory Cytokines and Chemokines

**Figure 4:**
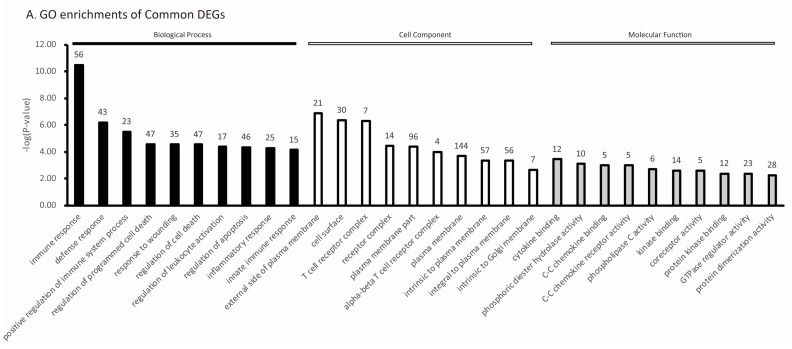

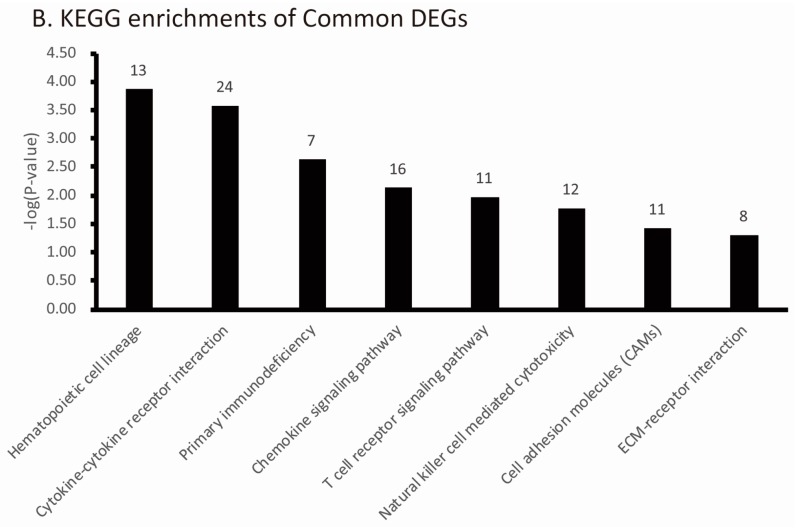
The Top Ten gene ontology (GO) enrichments and Kyoto Encyclopedia of Genes and Genomes (KEGG) enrichments of common DEGs. (**A**) The Top Ten GO enrichments of DEGs in biological process, cell component and molecular function of Common; (**B**) the KEGG enrichments of common DEGs. The X-axis is the name of each category, the Y-axis is their −log (*p* value). The number of genes enriched in each category were shown at the top of each bar.

To reveal the host’s response to PRRSV infection, the common enriched terms were compared. We submitted all the DEGs corresponding to PRRSV infection (1806 for TC pigs, 2155 for LW pigs) to DAVID for GO analysis, which identified 124 common enriched biological process terms for both breeds (Table S1). The top 10 significantly enriched terms sorted by *p* value in each functional category are shown in Figure 4A, among which the enriched biological processes (BP) were related to immune response and cell death regulation. The KEGG pathway analysis identified eight significant signaling pathways with a *p* value < 0.05 (Figure 4B), among which the cytokine–cytokine receptor interaction (including *CXCR3*, *CXCR6*, *CCR4*, *CCR5*, *CCR7*, *CCR9*), chemokine signaling (including *Src*, *Tiam1*, *PLCβ*, *RasGRP2*), T cell receptor signaling (including *CD247*, *CD3d*, *CD3e*, *CD3g*, *CTLA4*, *LCK*, *PLG2*) and natural killer (NK) cell mediated cytotoxicity (including *FASLG*, *SH2D1A*, *SH2D1B*, *GZMB*, *TNFSF10*, *ZAP70*) were directly related to infection and immune response. We submitted the common DEGs to the ingenuity pathway analysis (IPA), which identified lipopolysaccharide, *TGFB1*, *TNF*, *TCR* and *IFN-gamma* as the top 5 upstream regulators.

### 2.5. The Specific Enrichments of TC and LW Pigs’s DEGs

To elucidate the response difference between TC and LW pigs, we submitted all DEGs to DAVID. Besides the common enriched terms, 89 and 84 specific terms for TC and LW pigs of GO functional category analysis were also identified (Table S1). For TC pigs, three terms (GO:0070482, GO:0033189 and GO:0055114) of the top ten significantly enriched terms were related to oxidation, including the DEGs such as *BCL2*, *EGLN3*, *EDN1*, *TFRC*, *VEGFA*. Besides, two terms (GO:0050729 and GO:0002861) were related to inflammatory response, including *FCER1G* and *PRKCA*. In addition, nine cell proliferation related terms (GO:0042129, GO:0048660, GO:0008284, GO:0042130, GO:0008283, GO:0050671, GO:0032946, GO:0070665 and GO:0048661) were highlighted within TC specific GO term list, involved in 64 DEGs including *CCL2*, *CXCL10*, *TNFRSF11A*, *TNFRSF17*, *TNFRSF4*, *CSF1R*, *VEGFA*, *IL7R*, *IL18*, *IL15RA* and *IFN-γ*. Within the LW pig’s specific terms, seven of the top ten significantly enriched terms were related to lipid metabolism and transportation (including *FABP1*, *PPARG*, *PLTP*, *LIPG*, *SLC26A1* and *SLC26A6*). Besides, five cytotoxicity related terms (GO:0001910, GO:0001909, GO:0001912, GO:0002858 and GO:0002860) were highlighted within LW specific GO term list, involved in 10 DEGs, such as *CD226* and *NECTIN2*.

The pathway analysis based on the KEGG database revealed that TC pigs specifically enriched in biosynthesis of amino acids (especially arginine) and cell adhesion molecules (including *CD80*, *CD86*, *CLDN*, *ITGB1* and *ITGB7*). LW pigs had more specific enriched pathways, including TNF signaling pathway (including *VCAM1*, *IL1B1*, *CXCL2*, *CXCL10*, *CCL2*, *CCL5*, *CCL20*, *TNFAIP3*), lysosomal pathway, NOD-like receptor signaling pathway, PPAR signaling pathway (including *CD36*, *ACAA1*, *ACSL1*, *ACSL5*, *ACOX2*, *FABP3*, *PPARG*, *PLIN1*) (Table S2).

All DEGs were submitted to the IPA software, yielding top 30 canonical pathways of two breeds were listed separately in Table 2, of which17 pathways were shared by two breeds (Table 2). Among them, eleven predicted pathways were activated and two predicted pathways were inhibited after infection.

### 2.6 Comparison with the Published Data

**Figure 5:**
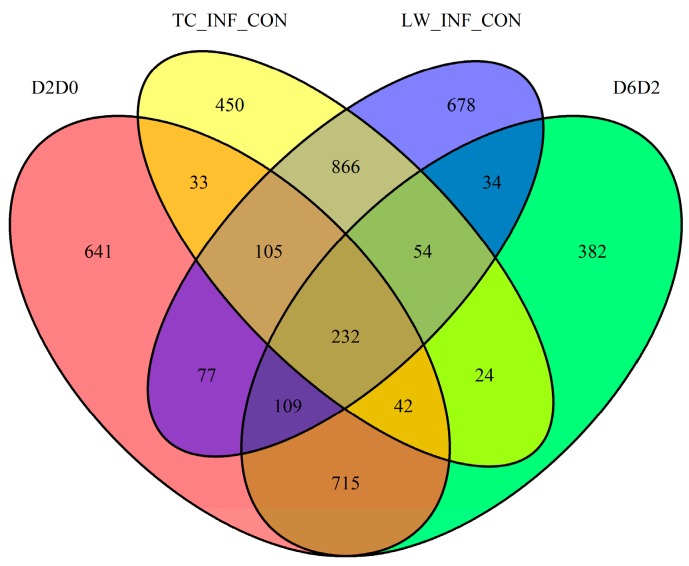
The Venn Diagram of DEGs distribution in this research and published data. The numbers in overlapping areas represent DEGs shared among the groups.

**Figure 6:**
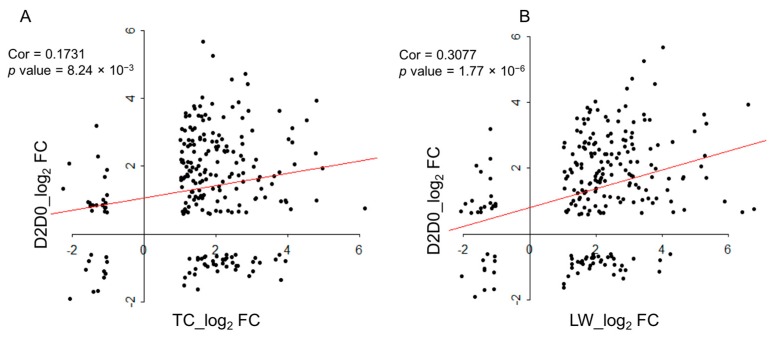
The scatterplots of the log_2_ (Fold change) of the common DEGs of TC_INF_CON (**A**) or LW_INF_CON (**B**) with D2D0. The X-axis is the DEGs’ log_2_ (Fold change) of TC_INF_CON/LW_INF_CON and the Y-axis is the log_2_ (Fold change) of D2D0. The correlation coefficient and *p* value is marked.

Jamie et al. reported a time-course transcriptome analysis during the first week after type 2 PRRSV infection [19], and the DEGs of 2 dpc vs. 0 dpc (D2D0) and 6 dpc vs. 2 dpc (D6D2) were identified. We compared the DEGs of TC_Infection vs TC_Control groups (TC_INF_CON) and the DEGs of LW_Infection vs. LW_Control groups (LW_INF_CON) with published DEGs, and the enrichment analysis was conducted as well. There were 232 common DEGs among the four groups, 105 DEGs among TC_INF_CON, LW_INF_CON and D2D0, and 54 DEGs among TC_INF_CON, LW_INF_CON and D6D2 (Figure 5). For the 232 common DEGs, we further analyzed the fold change correlations between D2D0 and TC_INF_CON and LW_INF_CON respectively (Figure 6). It revealed that the 232 common DEGs in both TC and LW pigs displayed significant positive correlations with that in D2D0 under PRRSV infection, indicating a high accordance of our present data and the published data.

Three hundred and ninety-one common DEGs were uploaded to DAVID as important candidate genes responding to PRRSV infection for enrichment analyses (Table S3). Among the top 10 biological process terms in GO enrichment, most were about immune response, such as defense response to virus (GO:0051607), negative regulation of viral genome replication (GO:0045071) and neutrophil chemotaxis (GO:0030593). The KEGG pathway analysis revealed the enriched terms, including T cell receptor signaling pathway (count = 10, *p* value = 4.48 × 10^−4^), *RIG-I* like receptor signaling pathway (count = 6, *p* value = 1.55 × 10^−2^), and antigen processing and presentation (count = 7, *p* value = 2.89 × 10^−4^).

## 3. Discussion

The emergence of PRRSV has garnered widespread attention due to its devastating impacts on the swine industry. As a result, researchers have studied PRRSV through the comparison of pathogenesis, viral load, cytokine production, clinical syndromes, and transcriptome analysis in pig breeds. In the current study, RNA-Seq technology was used to compare transcriptome differences of PAMs between TC and LW pigs.

With the common DEGs, the host’s immune response to PRRSV was observed. During the PRRSV infection, the host pathogen recognition receptors (PRRs) were engaged in the antiviral response. The analysis suggested that cytoplasmic PRRs *DDX58* (retinoic-acid-inducible protein I, *RIG-I*) and melanoma differentiation-associated gene 5 (*MDA5*) were expressed at high levels after infection, as well the cell surface PRRs such as *CD14*. But the Toll-like PRRs molecules were not activated. PRRs were initiated leading to intracellular signaling cascades that activated transcription factors such as *IRF7*. The activation of PRRs might have stimulated the production of inflammatory cytokines and chemokines, which would help recruit immune cells. Many up regulated genes, such as *selectin L* (*SELL*), *integrin* α 2 (*ITGA2*), *ITGA3*, *ITGA6*, *ITGAE* and *integrin* β *8* (*ITGB8*) were also believed to play a role in the recruitment of immune cells. The immune cells commonly recruited in this process included T cells, B cells and NK cells. Several T cells surface receptor (including *CD8* and *CD3*) were up regulated to help stimulate cytokine release. The comparison with the published data further confirmed these enrichments.

**Figure 7:**
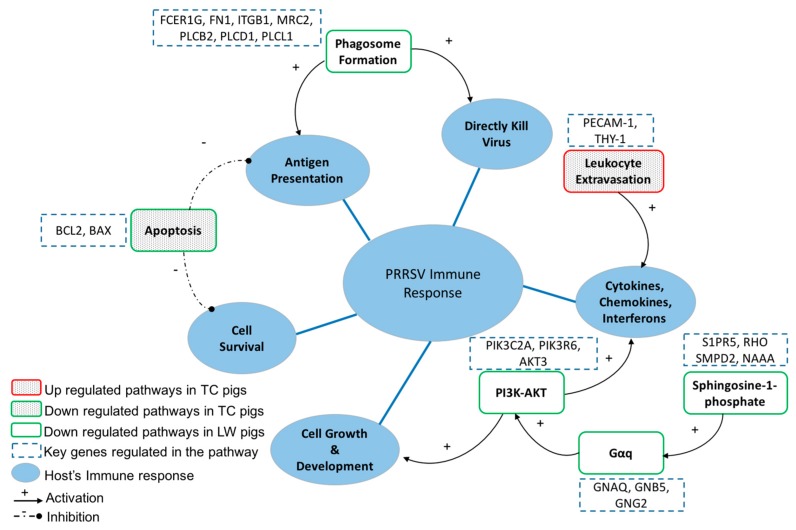
The induction of Porcine reproductive and respiratory syndrome virus (PRRSV) immune response in TC and LW pigs. There were five sorts of host immune response found in the current study: directly killing virus, blocking antigen presentation, prolonging cell survival, promoting cell growth and development/secretion of cytokines, chemokines and interferons. Pathways enriched in TC or LW pigs were distinguished with different filling. Upregulated pathways were marked with a red box and downregulated pathways are marked with a green box. The activation or inhibition role to downstream pathways were represented with “+” or “−”. Key DEGs in each pathway were shown in their nearby dash line box.

Based on the specific enrichments, the response differences between TC and LW pigs were compared. According to their specific enriched IPA terms, we classified the pathways that the host used to defend against PRRSV infection into five categories, which include directly killing virus, blocking the antigen presentation, prolonging cell survival, promoting cell growth and development/secreting cytokines, chemokines and interferons to activate downstream pathways (Figure 7). These five categories may provide a reference regarding hosts’ immune response to PRRSV.

During leukocytes recruitment into the sites of infection, the leukocytes will migrate through spaces between endothelial cells with the help of pseudopodia [20] and will secrete the proteases like *MMP-9* to degrade the basement membrane, which allows the leukocytes to enter into the infected tissue [21]. In our dataset, the leukocyte extravasation signaling was specifically activated in TC pigs and belonged to the top 30 canonical pathways (molecular = 25, −log_10_ (*p* value) = 3.97) (Figure S1). Among these molecules, the immunoglobulin supergene family members, *PECAM1* and *THY1* were upregulated. Ig-domains of *PECAM1* was required for the migration of monocytes through endothelial cells joint [22] and *THY-1* mediates the adhesion of granulocytes and monocytes to the activated endothelial cells and this interaction has an essential role in the control of the migration of granulocytes and monocytes from blood into the tissues during inflammation or infection [23]. The activation of leukocyte extravasation signaling in TC pigs might enable them with the ability to defend themselves against various pathogens. Moreover, several chemokines that are required in the activation of this signal, including *CXCR3*, *CXCR6*, *CXCL10*, *CXCL11* and *CXCL17*, were also consistently upregulated in TC pigs.

Apoptosis is a highly regulated process modulated by both pro-apoptotic and anti-apoptotic cellular factors [24,25]. The competition of cell death inhibitors (such as *BCL-2*) and cell death inducers (such as *BAX*) desires the outcome of apoptosis [26,27]. In the present study, both apoptosis suppressive genes, *BCL-2* (log_2_ FC=1.220, *p* value = 0.0053) and *BCL-XL* (log_2_ FC = 1.365, *p* value = 5.00 × 10^−5^), were upregulated in TC pigs during PRRSV infection, while they were not changed in PRRSV infected LW pigs. The Western-blot assay result further identified that TC had more *BCL-2* expression and less *pro-casp3* expression. Given that TC pigs had a much lower viral load than LW pigs during 1–7 dpc [17], we speculate that the inhibition of apoptosis during early infection stage may offer more time for the infected macrophages to present viral antigens to T cells, which may be an important strategy for TC pigs to control PRRSV replication by adaptive immunity.

The phagosome formation signal is also an essential pathway for the host to kill the pathogens and present the antigens to the immune system [28]. Some viruses that are phagocytized by macrophages and polymorphonuclear leukocytes will be killed, and the remaining viral particles from the phagosomes may be transported to the endocytic pathways for further processing and binding to *MHC* molecules [29]. In the present study, we observed the downregulation of phagocytic cell receptors in both breeds, including integrin receptor (*ITGB1*, *FN1*) and macrophage mannose receptor (*MRC2*), as well as some of their downstream effectors (*PIK3C2A*, *PIK3R1*, *PIK3R6*, *PLCB2*, *PLCD1*, *PLCL1*) (Figure S2). This likely means that, PRRSV, a highly pathogenic virus, will also suppress phagocytic response and avoid the uptake by the host cells. Nevertheless, LW pigs displayed stronger suppressive effect than TC pigs. In addition, the macrophage receptor coding gene *MARCO* (log_2_ FC = −2.484, *p* value = 5.00 × 10^−5^) and the toll-like receptor coding gene *TLR8* (log_2_ FC = −1.259, *p* value = 5.00 × 10^−5^) were specifically inhibited in LW pigs. Additionally, the genetic basic comparison revealed that TC pigs had a higher expression among *MHC* genes than LW pigs, indicating their stronger capacity of antigen presentation and processing. Thus, the lower viral load observed in TC pigs probably has a close relationship to the phagosome formation signaling.

**Figure 8:**
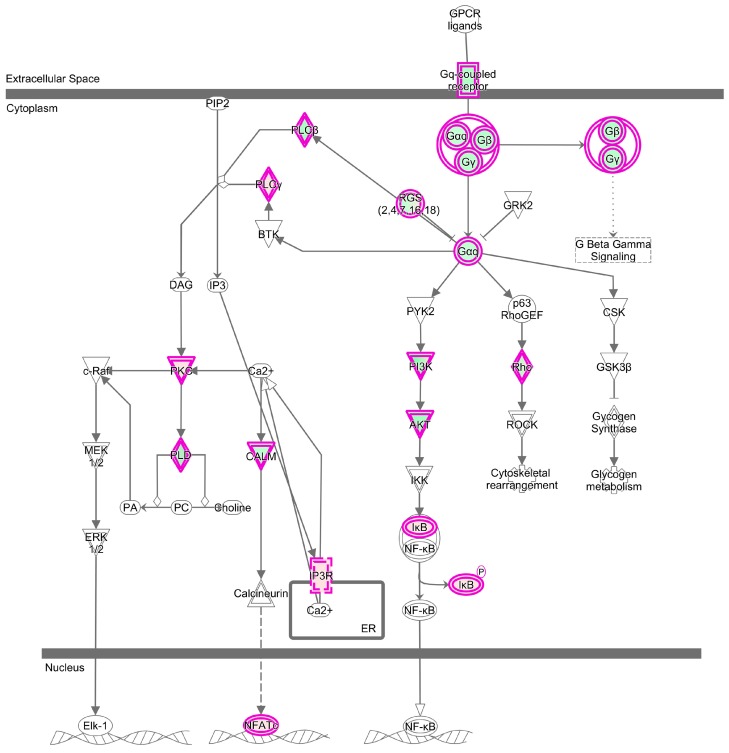
G-protein signaling of LW pigs. The DEGs enriched in this pathway were marked with fuchsia frame, upregulated genes were filled with pink and downregulated genes were filled with green.

The heterotrimeric G-proteins are signaling molecules that transduce extracellular signals, such as chemokines and neurotransmitters, to downstream signaling molecules, including *PLCβ*, *PI3K* and *Rho* [30,31], which play essential roles in the regulation of host immune response. Neither the Gq-coupled receptor nor the G protein subunits had any change in the PRRSV infected TC pigs, while three *Gαq* signaling related DEGs were identified in PRRSV infected LW pigs, including *GNAQ* (log_2_ FC = −1.040, *p* value = 4.00 × 10^−4^), *GNB5* (log_2_ FC = −1.347, *p* value = 5.00 × 10^−5^) and *GNG2* (log_2_ FC = −1.291, *p* value = 5.00 × 10^−5^). As a result, its downstream signaling pathway, phosphatidylinositol 3-kinase (*PI3K*)-serine-threonine kinase (*AKT*) (including *PIK3C2A*, *PIK3R1*, *PIK3R6*, *AKT3*), was also downregulated (Figure 8). Besides, CALM (*CALM4*, ENSSSCG00000023497, log_2_ FC = −1.200, *p* value = 5.00 × 10^−5^) and Rho (*RHOQ*, ENSSSCG00000008439, log_2_ FC = −1.358, *p* value = 5.00 × 10^−5^) were also inhibited in LW pigs. The enzyme *PI3K* is central to many cell signal transduction pathways including cell growth, survival, death and cycle progression. It acts on several downstream effectors, including *AKT*, to play a role in these events [32,33]. The inhibition of *Gαq* signaling (*GNAQ*, *GNB5*, *GNG2*) in LW pigs induced the inhibition of *PI3K-AKT* signaling (*PIK3C2A*, *PIK3R1*, *PIK3R6*, *AKT3*), and as a result, its downstream signalings, including cell cycle progression, cell death, cell growth, cell survival and macrophage survival, were inactivated. These findings may, in some extent, explain why LW had a weaker immune response to PRRSV infection than TC pigs. Additionally, compared the DEGs with the published data in Jamie’s article, we found that G-protein signaling was also downregulated in their data. It not only indicated good accordance of both research, but also suggested that the suppression of G-protein signaling maybe a strategy for PRRSV to suppress host’s immune response.

The sphingosine 1-phosphate (*S1P*) is an important lipid mediator that has been implicated in many biological processes, such as cellular proliferation and survival [34]. Five receptors have been reported, *S1P1*, *S1P2*, *S1P3*, *S1P4* and *S1P5*. These receptors are ubiquitously expressed and coupled to a variety of G proteins, including *Gαq*, *Gαi* and *Gα 12/13* [35]. In our database, the catalyzing proteins of ceramide (*SMPD2*, ENSSSCG00000004408, log_2_ FC = −1.031, *p* value = 5.00 × 10^−5^) and sphingosine (*NAAA*, ENSSSCG00000008973, log_2_ FC = −2.501, *p* value = 5.00 × 10^−5^; *SGPP1*, ENSSSCG00000028558, log_2_ FC = −1.252, *p* value = 5.00 × 10^−5^) were both downregulated in LW pigs (Figure S3). These may weaken the immune response to infection. Furthermore, the receptor *S1P5* (ENSSSCG00000028460, log_2_ FC = −1.690, *p* value = 0.0436) and its downstream gene *Rho* (*RHOQ*, ENSSSCG00000008439, log_2_ FC = −1.358, *p* value = 5.00 × 10^−5^) were also inhibited. Even though the other four receptors were not suppressed, their coupled protein *Gαq* and the effector genes of *Gαi* were downregulated. Altogether, as an upstream pathway, the inhibition of the S1P signaling in LW pigs may influence some downstream pathways and weaken the immune response to the PRRSV infection.

## 4. Materials and Methods

### 4.1. PRRSV Artificial Challenge and Sample Collection

A total of twenty-four 5-week-old piglets (12 TC pigs and 12 LW pigs) were randomly chosen to perform the artificial challenge experiment as described previously [4]. Six TC pigs and six LW pigs received an intramuscular challenge at a viral dose of 10^5^ CCID_50_/mL (3 mL/15 kg), and the rest control pigs were challenged with the same amount of RPMI-1640 (Gibco, Grand Island, NY, USA). All pigs were humanely euthanized for necropsy at 7 dpc. All the left lungs were collected and the phosphate buffer was used to perform the bronchoalveolar lavage with conventional method [36]. The filtrations of bronchoalveolar lavage fluid were resuspended with Trizol reagent (Invitrogen, Carlsbad, CA, USA) for total RNA extraction from PAMs as the manufacturer’s protocol.

All animal procedures were approved by the Ethical Committee for Animal Experiments at Huazhong Agricultural University, Wuhan, China. The animal experiments were performed at the Laboratory Animal Center of Huazhong Agricultural University (Animal experiment approval No. HZAUSW-2013-005, 08/27/2013).

### 4.2. RNA Preparation and Sequencing

RNA degradation and contamination was monitored on 1% agarose gels and RNA purity, concentration, and integrity were measured by Nanodrop, Qubit Fluorometer and Agilent 2100 Bioanalyzer. The total RNA of PAMs from 12 selected pigs (3 pigs selected randomly from each group) were used for sequencing with NEBNext^®^ UltraTM RNA Library Prep kit for Illumina^®^ (NEB, Ipswich, MA, USA), and all the procedures and standards were performed following the manufacturer’s protocols. After library preparation and quality control, the library preparations were sequenced on an Illumina Hiseq 2000 platform and 100 bp paired-end reads were generated.

### 4.3. RNA-Seq Data Analysis and DEGs Calling

Clean reads were obtained by removing reads containing adapter or poly-*N* and low quality reads from raw reads. Clean reads were aligned against ENSEMBL Suscrofa10.2.72 with bowtie v2.1.0 (available online: http://bowtie-bio.sourceforge.net/index.shtml) and TopHat v2.0.9 (available online: https://github.com/infphilo/tophat) with the option --library-type fr-first strand. Cufflinks, Cuffcompare and Cuffmerge scripts from Cufflinks v2.1.1 (available online: https://github.com/cole-trapnell-lab/cufflinks) package were used to assemble and merge transcripts. The Cuffdiff function was used to estimate the count variances of each transcript. Then gene expression was estimated and FPKM value was calculated. Genes would be removed if its FPKM quality status was not “OK” or the FPKM in all samples were zero. Cuffdiff also provided differential expression statistics of experimental and control groups. The *p* values were adjusted using the Benjamini & Hochberg method. Corrected *p* value of 0.05 and log_2_ (Fold change) (log_2_ FC) of 1 were set as the threshold for the identification of differentially expressed genes.

### 4.4. Significant Functions and Gene Network Analysis

The differentially expressed genes were sorted by the enrichment of GO categories and KEGG database in David. The human homologous Ensemble Gene IDs of differentially expressed genes were subjected to biological network, functional classification and pathway analyses using IPA. The networks and canonical pathway generated by IPA were established on its genes’ connectivity and were scored based on the hypergeometric distribution with the right-tailed Fisher’s exact test. The score was displayed as the negative log of this *p* value.

### 4.5. Validation of Differentially Expressed Genes with Quantitative Real Time RT-qPCR and Western-Blotting

RT-qPCR was performed with the total RNA from PAMs that were used in RNA-Seq with Oligo (dT) and random primers (Fermentas, ON, Canada). cDNA was used for RT-qPCR with SYBR Green PCR Master Mix, following the instruction manual. The reactions were performed on a Roche Lightcycle 480 Sequence Detection System. Triplicate wells of reactions (10 mL) contained 5 mL SYBR Green Master Mix, 1 mL of 50 ng/mL cDNA, 0.3 mL 10 mM of each primer and 3.4 mL ddH_2_O. The RT-qPCR conditions are 95 °C for 10 min, and then followed by 40 cycles (at 94 °C for 30 s, 60 °C for 30 s, and 72 °C for 10 s), and fluorescence acquisition at 72 °C in a single mode. The gene SDHA was chosen as the internal reference gene and the 2^−ΔΔ*C*t^ method was used to calculate the fold change for gene expression.

The protein was collected after centrifugation of PAMs suspended with RIPA lysis buffer (Biosharp, Hefei, China). The protein concentration was measured with Bradford protein assay kit (Tiangen, Beijing, China). The protein was separated on SDS-PAGE gel electrophoresis and transferred to polyvinylidene diuoride membranes. After blocking, the membranes were incubated in the primary antibodies (Caspase 3 (ab4051, Abcam, Cambridge, UK), BCL-2 (ab117115, Abcam, Cambridge, UK), β-actin (AA128, Beyotime, Shanghai, China)) and in secondary antibodies (HRP-labeled Goat Anti-Mouse IgG(H+L) (A0216, Beyotime, Shanghai, China), HRP-labeled Goat Anti-Rabbit IgG(H+L) (A0208, Beyotime, Shanghai, China)), respectively. The resulting signals were visualized by ECL detection kit (Bio-Rad, Hercules, CA, USA). The results were analyzed using Image J software (available online: https://imagej.net).

## 5. Conclusions

Although only three individuals from each group were used, it still provides insights into a general trend and offers valuable data for DEGs and pathway analyses related to PRRSV infection and resistance. Overall, we identified 1179 upregulated and 627 downregulated genes in TC pigs, and 1154 upregulated and 1001 downregulated genes in LW pigs. Apart from the common enrichments, these two breeds had great differences in many signaling pathways. After PRRSV infection, TC pigs promoted leukocyte extravasation to stimulate the release of cytokines, which would accelerate the migration of immune cells to sites of infection and increase survival rate. The inhibition of apoptosis and activation of phagosome formation may aid in extending host cell survival, killing viral particles, and promoting antigen presentation to T cells. On the other hand, the suppression of S1P, Gαq and PI3K-AKT signaling in LW pigs may interfere with the host’s ability to establish an effective immune response against PRRSV infection and may explain why LW pigs had a higher viral load and lower survival rate.

## Figures and Tables

**Table 1:** Number of single-end 100 bp clean reads obtained and percentages of mapped reads per individual.

Group	Individual	Clean Reads	% Mapped Reads	Q20 (%)	Transcript Number (FPKM > 0.1)	Transcript Number (0.1 < FPKM < 1)
Tongcheng (TC)_Control	TC_C1	155,542,274	77.94	95.56	55,207	35,568
	TC_C2	144,420,088	76.08	95.93	72,765	54,856
	TC_C3	159,784,438	78.84	95.96	54,234	35,805
TC_Infection	TC_I1	150,738,786	72.96	95.97	56,554	37,232
	TC_I2	158,394,072	60.85	95.38	60,754	41,058
	TC_I3	147,835,638	76.54	96.45	54,838	35,665
Large White (LW)_Control	LW_C1	161,061,654	79.17	95.96	60,858	42,165
	LW_C2	110,922,288	77.54	95.70	75,926	56,881
	LW_C3	146,633,740	78.62	95.95	69,040	51,313
LW_Infection	LW_I1	158,938,676	72.97	95.65	73,211	54,774
	LW_I2	136,571,286	70.36	95.49	66,589	49,258
	LW_I3	140,256,430	75.98	95.92	72,837	54,384

**Table 2:** The top 30 canonical pathways of all DEGs in LW pigs and TC pigs

Category	LW	Count	−log_10_ (*p* Value)	TC	Count	−log_10_ (*p* Value)
Shared	Phospholipase C Signaling	40	7.29	Phospholipase C Signaling	37	7.886
	Hepatic Fibrosis/Hepatic Stellate Cell Activation	36	8.542	Hepatic Fibrosis/Hepatic Stellate Cell Activation	30	7.11
	Granulocyte Adhesion and Diapedesis	31	6.372	Granulocyte Adhesion and Diapedesis	27	5.923
	T cell Receptor Signaling	18	4.077	T cell Receptor Signaling	19	5.723
	Calcium-induced T Lymphocyte Apoptosis	13	3.455	Calcium-induced T Lymphocyte Apoptosis	15	5.596
	Role of Macrophage, Fibroblasts and Endothelial Cells in Rheumatoid Arthritis	39	4.564	Role of Macrophage, Fibroblasts and Endothelial Cells in Rheumatoid Arthritis	37	5.568
	Molecular Mechanisms of Cancer	42	3.508	Molecular Mechanisms of Cancer	42	5.281
	Phagosome Formation	21	5.265	Phagosome Formation	19	5.278
	CCR5 Signaling in Macrophage	15	4.201	CCR5 Signaling in Macrophage	15	5.114
	Agranulocyte Adhesion and Diapedesis	28	4.454	Agranulocyte Adhesion and Diapedesis	26	4.94
	CTLA4 Signaling in Cytotoxic T lymphocytes	16	3.848	CTLA4 Signaling in Cytotoxic T lymphocytes	16	4.78
	Reelin Signaling in Neurons	16	4.437	Reelin Signaling in Neurons	15	4.753
	NF-κB Activation by Virus	14	3.627	NF-κB Activation by Virus	14	4.458
	Pathogenesis of Multiple Sclerosis	5	3.952	Pathogenesis of Multiple Sclerosis	5	4.328
	LPS/IL-1 Mediated Inhibition of RXR Function	31	4.284	LPS/IL-1 Mediated Inhibition of RXR Function	27	4.081
	Caveolar-mediated Endocytosis Signaling	14	3.692	Caveolar-mediated Endocytosis Signaling	13	3.9
	HER-2 Signaling in Breast Cancer	14	3.501	HER-2 Signaling in Breast Cancer	13	3.717
Specific	Sphingosine-1-phosphate Signaling	23	6.142	Role of Tissue Factors in Cancer	19	5.1
	Gαq Signaling	24	4.254	VDR/RXR Activation	15	4.753
	Differential Regulation of Cytokine Production in Intestinal Epithelial Cells by IL-17A and IL-17F	8	4.225	Cytotoxic T Lymphocyte-mediated Apoptosis of Target Cells	9	4.475
	Thrombin Signaling	28	4.209	Role of Pattern Recognition Receptor in Recognition of Bacteria and Viruses	19	4.405
	Role of JAK1 and JAK3 in γC Cytokine Signaling	13	3.875	Virus Entry via Endocytic Pathways	16	4.353
	VDR/RXR Activation	15	3.862	Micropinocytosis Signaling	13	4.231
	P70S6K Signaling	20	3.845	Role of Osteoblasts, Osteoblasts and Chondrocytes in Rheumatoid Arthritis	27	4.081
	Role of NFAT in Regulation of the Immune Response	25	3.746	CD28 Signaling in T Helper Cells	18	3.986
	Human Embryonic Stem Cell Pluripotency	21	3.648	Leukocyte Extravasation Signaling	25	3.971
	Tec Kinase Signaling	23	3.609	Signaling by Pho Family GTPases	27	3.733
	Type I Diabetes Mellitus Signaling	18	3.595	IL-8 Signaling	23	3.692
	Glioma Invasiveness Signaling	12	3.564	HGF Signaling	16	3.673
	Colorectal Cancer Metastasis Signaling	30	3.381	p53 Signaling	15	3.638

## Supplementary Materials

Supplementary materials can be found at www.mdpi.com/1422-0067/18/7/1475/s1.

## Abbreviations

PRRS	Porcine Reproductive and Respiratory Syndrome
PRRSV	Porcine Reproductive and Respiratory Syndrome Virus
TC	Tongcheng
LW	Large White
PAMs	Porcine Alveolar Macrophages
DEGs	Differentially Expressed Genes
HR	High Response
LR	Low Response
ACHE	Acetylcholinesteras
XIST	X (inactive)-Specific Transcript
PHGC	PRRS Host Genetics Consortium
IFN	Interferon
TNF	Tumor Necrosis Factor
IL	Interleukin
MHC	Major Histocompatibility Complex
DPL	Dapulian
DLY	Duroc × Landrace × Yorkshire
FPKM	Fragments per Kilobase of Exon per Million Fragments Mapped
GO	Gene Ontology
BP	Biological Processes
IPA	Ingenuity Pathway Analysis
PRRs	Pathogen Recognition Receptors
