# Supplementary material for: Transcriptome Differences in Porcine Alveolar Macrophages from Tongcheng and Large White Pigs in Response to Highly Pathogenic Porcine Reproductive and Respiratory Syndrome Virus (PRRSV) Infection
Source: Int J Mol Sci. 2017 Jul 12;18(7):1475. doi: 10.3390/ijms18071475 (PMC5535966; doi:10.3390/ijms18071475)
Supplement: Supplementary file 1 [file ijms-18-01475-s001.zip › ijms201160-supplementary.pdf]

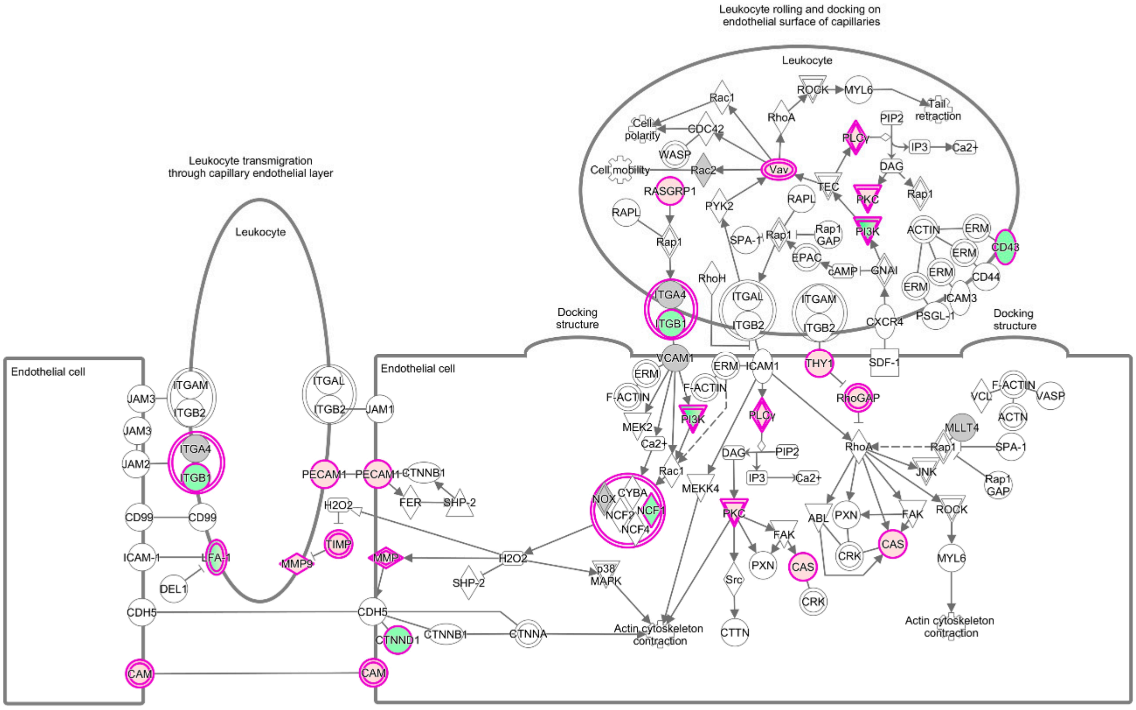

Figure S1. Leukocyte extravasation signaling of TC pigs. The DEGs enriched in this pathway were marked with fuchsia frame, upregulated genes were filled with pink and downregulated genes were filled with green.

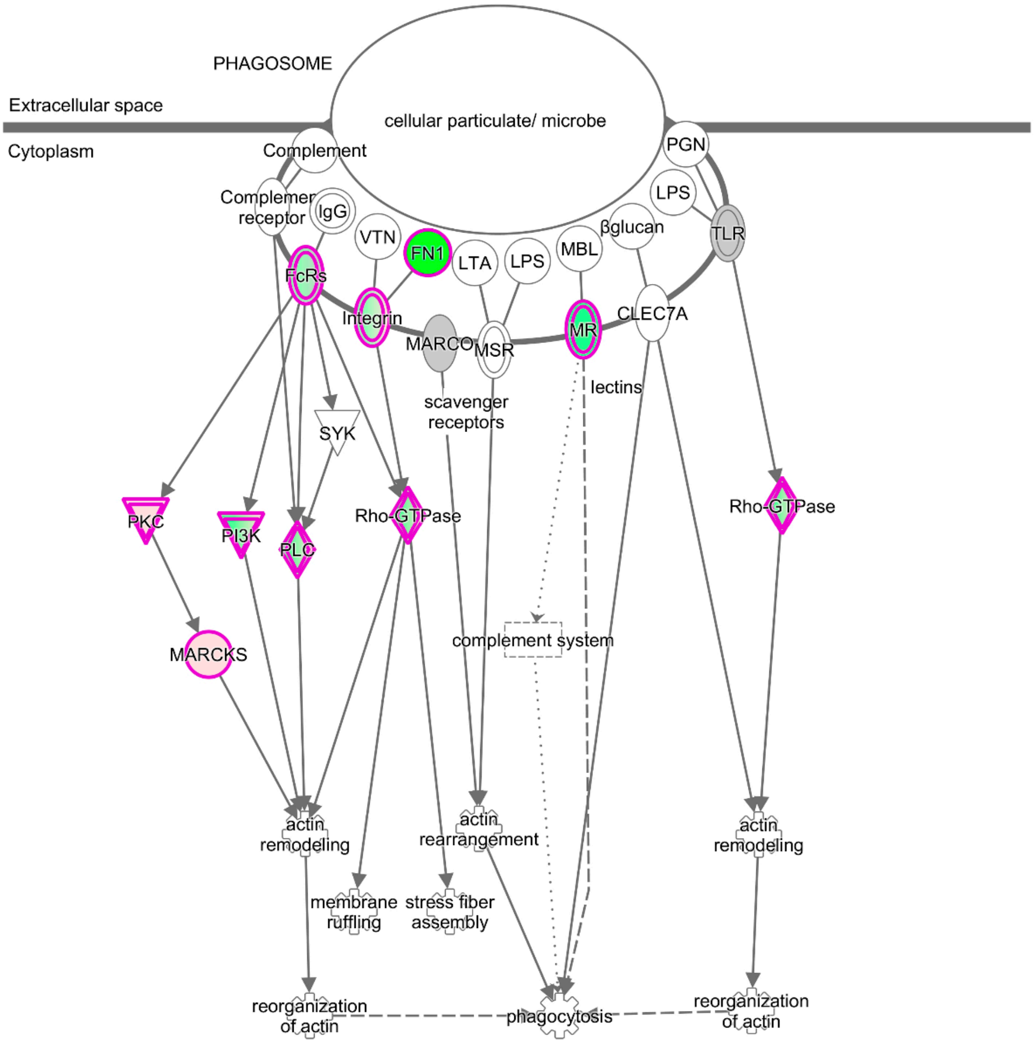

Figure S2. Phagosome formation signaling of TC pigs. The DEGs enriched in this pathway were marked with fuchsia frame, upregulated genes were filled with pink and downregulated genes were filled with green.

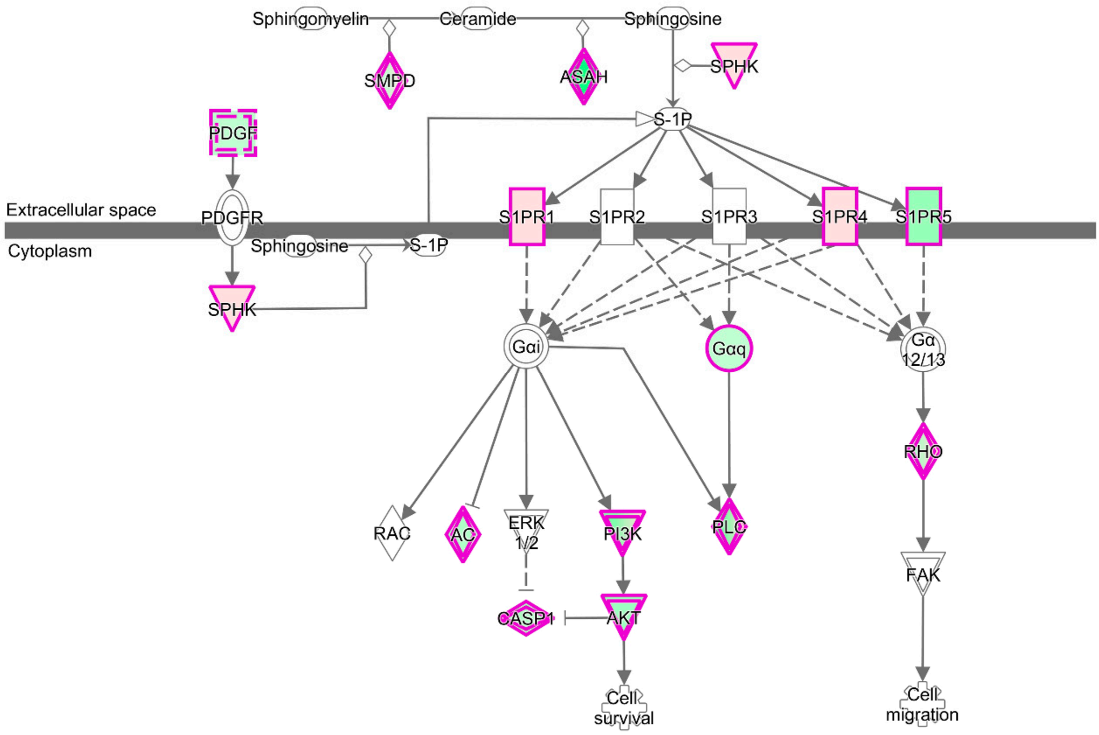

Figure S3. Sphingosine-1-phosphate Signaling. The DEGs enriched in this pathway were marked with fuchsia frame, upregulated genes were filled with pink and downregulated genes were filled with green.

Table S1. The specific GO enrichments of TC and LW pigs. The sheet "common" showed the specific genes in the common enriched terms between TC and LW pigs, the sheet "TC_Specific" and "LW_Specific" showed the specific GO terms enriched in TC and LW pigs.

Table S2. The KEGG pathway enrichment of all DEGs of TC and LW pigs.

Table S3. The DAVID enrichment of the common DEGs of TC_INF_CON, LW_INF_CON, D2D0, and D6D2.
